# In Vivo Imaging of Leukocyte Recruitment to the Atheroprone Femoral Artery Reveals Anti-Inflammatory Effects of Rosuvastatin

**DOI:** 10.1155/2013/962369

**Published:** 2012-12-30

**Authors:** Mizuko Osaka, Sumihiko Hagita, Masayuki Yoshida

**Affiliations:** ^1^Department of Life Sciences and Bioethics, Graduate School of Medical and Dental Sciences, Tokyo Medical and Dental University, 1-5-45 Yushima, Bunkyo-ku, Tokyo 113-8519, Japan; ^2^Department of Geriatrics and Vascular Medicine, Graduate School of Medical and Dental Sciences, Tokyo Medical and Dental University, 1-5-45 Yushima, Bunkyo-ku, Tokyo 113-8519, Japan

## Abstract

*Objective*. To monitor the anti-inflammatory effect of rosuvastatin in leukocyte endothelial interactions in the atheroprone femoral artery in vivo. *Methods and Results*. Male Apolipoprotein E null mice (ApoE−/− mice, 6 weeks old) were fed a high-fat diet (20% fat, 1.25% cholesterol) with or without the HMG CoA reductase inhibitor rosuvastatin (10 mg/kg/day) for 6 weeks. Significant leukocyte adhesion was observed in the femoral artery of ApoE−/− mice, but not of wild type mice, in the absence of rosuvastatin. Interestingly, no obvious plaque formation was observed in the artery at this time point. The number of adherent leukocytes was dramatically diminished in ApoE−/− mice treated with rosuvastatin. DHE-associated oxidative stress and the expression of gp91-phox, a component of NADPH oxidase, were induced in ApoE−/− mice and were abolished by rosuvastatin treatment. *Conclusion*. Our data documented leukocyte recruitment prior to lipid accumulation and subsequent inhibition by rosuvastatin. The underlying mechanism seemed to involve oxidative stress and an anti-inflammatory effect on the endothelium of atheroprone vessels.

## 1. Introduction

Inflammatory cascades, such as leukocyte recruitment to the vascular wall, play an important role in the development of atherosclerosis [1–3]. Recent clinical studies have suggested a positive correlation between the serum level of inflammatory markers and the rate of cardiovascular events [4, 5]. Careful pathological examination of atherosclerosis specimens have revealed the presence of inflammatory cells, such as monocytes, macrophages, and lymphocytes, at the lesion area, which are thought to be causatively involved in the development of atherosclerosis. Nevertheless, there is no direct evidence to confirm that monocyte recruitment to the luminal surface of the artery occurs prior to the lipid deposition in vivo. As previously demonstrated, leukocyte endothelial interaction in vivo is heavily influenced by local shear stress created by blood flow [6, 7], and thus it is intriguing to note that leukocyte recruitment is occurring at the arterial wall in the presence of relatively high shear stress without mechanical injury.

To investigate this issue, we developed a novel imaging system to dynamically visualize leukocyte recruitment to the femoral artery in vivo. Using this technique, we previously documented that mechanical injury to the arterial intima significantly induces leukocyte adhesion to the vascular wall [8]. 

In this study, we tried to demonstrate leukocyte adherence to the vascular wall in the absence of mechanical vascular injury in vivo and potential modulation by rosuvastatin treatment.

Statins are used clinically to reduce serum cholesterol levels leading to the reduction of cardiovascular events [9–11]. Several experimental studies, including ours, have indicated an anti-inflammatory role of statins in vitro [12, 13]. A recent clinical study has suggested a pleiotropic anti-inflammatory property for rosuvastatin which may play a role in reducing cardiovascular events among those with high serum CRP but normal cholesterol levels [14]. Though experimental animal studies have revealed that statins can inhibit or even diminish the development of atherosclerosis [15, 16], their effect on leukocyte-endothelial interactions in the development of atherosclerosis has not been demonstrated in vivo. Therefore, we sought to document recruitment of leukocytes at the vasculature of athero-prone animals in the absence of mechanical injury and the reversal of this recruitment by rosuvastatin.

## 2. Materials and Methods

### 2.1. Animals

C57BL/6 (6 weeks of age, male) mice were obtained from Charles River Laboratories Japan, Inc. and used as control (wild type: wt). Apolipoprotein E-deficient mice (6 weeks of age, male; ApoE−/− mice) were used in this study. They were provided with diet and water ad libitum. The experiments adhered to the APS Guiding Principles in the Care and Use of Animals and were approved by the Ethical Committee for Animal Experimentation of Tokyo Medical and Dental University. 

### 2.2. Intravital Microscopy (IVM)

Intravital microscopy (IVM) of the femoral arteries was performed on ApoE−/− mice fed normal chow (NC; CE-2, CLEA Japan, Inc., Tokyo) or high fat diet (HF; 1.25% cholesterol, 20% fat in CE-2; CLEA Japan, Inc., Tokyo) for 0, 2, or 6 weeks and was compared with those performed on wt mice. In some of those experiments, rosuvastatin (rosuva; 10 mg kg^−1^ per day, AstraZeneca KK) or vehicle (water) was simultaneously administered to ApoE−/− mice fed HF and leukocyte adhesion was observed in the femoral artery as previously detailed [8]. In brief, mice were anesthetized with pentobarbital and mechanically ventilated so as to maintain a normal acid-base balance. Rectal temperature was maintained at 36.0-37.0°C with a heating pad and an infrared heat lamp. After injection of Rhodamine 6G chloride (Invitrogen, Carlsbad, CA, USA; 0.3 mg kg^−1^ in 300 *μ*L of phosphate buffered saline (−)) into the right femoral vein, the left femoral artery at the level of the epigastric branch was visualized with a fluorescent microscope (BX51WI, Olympus, Tokyo, Japan) equipped with a water immersion objective (×20). Epifluorescence was illuminated by a 100-W fluorescent lamp source and images were directly captured with a PC via a CCD camera (CoolSnap HQ, Olympus). Adhesion of labeled leukocytes was clearly visualized on the anterior half of the vessels facing the objective. All images were recorded using a computer-assisted image analysis program (Meta Morph) according to the manufacturer's protocol. The parameters used to characterize the adhesive interactions of leukocytes have been described in detail previously [17]. The number of adherent leukocytes (i.e., those that did not move for ≥3 s during the 1 min recording period) was counted along a region of interest (ROI), a 10^4^ *μ*m^2^ segment of the vessel and expressed as the number of adherent cells per 10^4^ *μ*m^2^ of the vessel surface. Image analysis was carried out as previously described [8, 18–22]. 

### 2.3. In Vivo Blockade of VCAM-1 in ApoE−/− Mice Fed HF

For blockage of VCAM-1, we intravenously injected 0.1 mg of anti-mouse VCAM-1 antibody (purified rat anti-mouse CD106 (VCAM-1), clone: 429 (MVCAM.A), #553330; BD Pharmingen) or control isotype IgG (purified rat IgG2a, *κ* isotype control, clone: R35-95. #553927) (*n* = 3) to ApoE−/− mice fed HF for 6 weeks by tail vein [23–25], and performed IVM at 30 min after antibody injection. The total number of leukocytes recruited to the vessel wall was derived from the sum of adherent and rolling cells.

### 2.4. Lipid Analysis

The total cholesterol (TC) and triglyceride (TG) levels in the plasma were measured after IVM with Seiken T-CHO (Denkaseiken Co. Ltd.) and *E*-test Wako (Wako Co. Ltd.), respectively. 

### 2.5. Quantification of Oxidative Stress in Aortas

To detect the change in the oxidant stress in the vessel wall of each group, staining of the aortas with dihydroethidium (DHE) was performed. Nonfixed aortas were cut into sections of 4 mm (in thickness). The sections were incubated with 5 *μ*mol/L DHE solution for 10 min at 37°C followed by observation under fluorescent microscopy. Mean fluorescence intensity (MFI) of 3 segments in each aorta was measured with IPLab imaging software (BioVision Technologies, Inc.).

### 2.6. Quantitative Real-Time PCR

The aortas of each group fed HF or NC for 6 weeks were harvested, and total RNA was extracted using RNeasy mini kit (QIAGEN). The first-strand cDNA was synthesized with a commercial reverse transcriptase (Invitrogen). Quantitative PCR was performed using with a 7900 Real-Time PCR System using the manufacturer's recommended protocol (Applied Biosystems, Foster City, CA, USA) with the FastStart Universal SYBR Green Master (Roche). The oligonucleotide primers for the experiments were as follows: beta-actin; 5′-AGCCATGTACGTAGCCATCC-3′ and 5′-CTCTCAGCTGTGGTGGTGAA-3′, p22-phox; 5′-TGGACGTTTCACACAGTGGT-3′ and 5′-TAGGCTCAATGGGAGTCCAC-3′, gp91-phox; 5′-ACTGCGGAGTTGGAAGA-3′ and 5′-GGTGATGACCACCTTTTGCT-3′, ICAM-1; 5′-AGCACCTCCCCACCTACTTT-3′ and 5′-AGCTTGCACGACCCTTCTAA-3′, VCAM-1; 5′-CCCGTCATTGAGGATATTGG-3′ and 5′-GGTCATTGTCACAGCACCAC-3′.

### 2.7. Oil Red-O Staining

After 16 weeks of treatment, ApoE−/− mice were euthanized, and lengthwise incision was made in each aorta. Cross-sections embedded in OCT compound were prepared from aortas or femoral arteries of ApoE−/− treated with rosuvastatin or vehicle for 16 weeks. The aortas were then washed twice with phosphate buffered saline (PBS) and fixed for 10 min with 10% formalin solution, followed by 60% isopropanol for 1 min. The specimens were stained with oil red O solution for 15 min, and the surface area of the atherosclerotic lesions was photographed. Similarly, cross-sections of femoral artery in ApoE−/− mice fed NC at age of 52 weeks was staining. 

### 2.8. Intravenous Injection of Externally Labeled MNCs

In some experiments, recipient or donor mice were administered HF with rosuvastatin or vehicle for 6 weeks. Mononuclear cells (MNCs) were isolated from peripheral blood taken from two ApoE−/− mice treated with rosuvastatin or vehicle by gradient centrifugation using an LSM (Histopaque-1083, Sigma Aldrich Corp., St Louis, MO, USA). They were labeled with Rhodamine 6G chloride and then 5 × 10^5^ cells were injected intravenously into the mice, and intravital microscopy was performed at 5 min after cell injection. 

### 2.9. Statistical Analysis

Data are expressed as the mean value ± s.e.m. One-way analysis of variance with a Tukey's *post hoc* test or two-tailed unpaired *t*-test was used to estimate statistical significance, with a value of *P* < 0.05 considered to be statistically significant. 

## 3. Results

### 3.1. Real-Time Observation of Leukocyte Recruitment to Femoral Artery in ApoE−/− Mice

**Figure 1:**
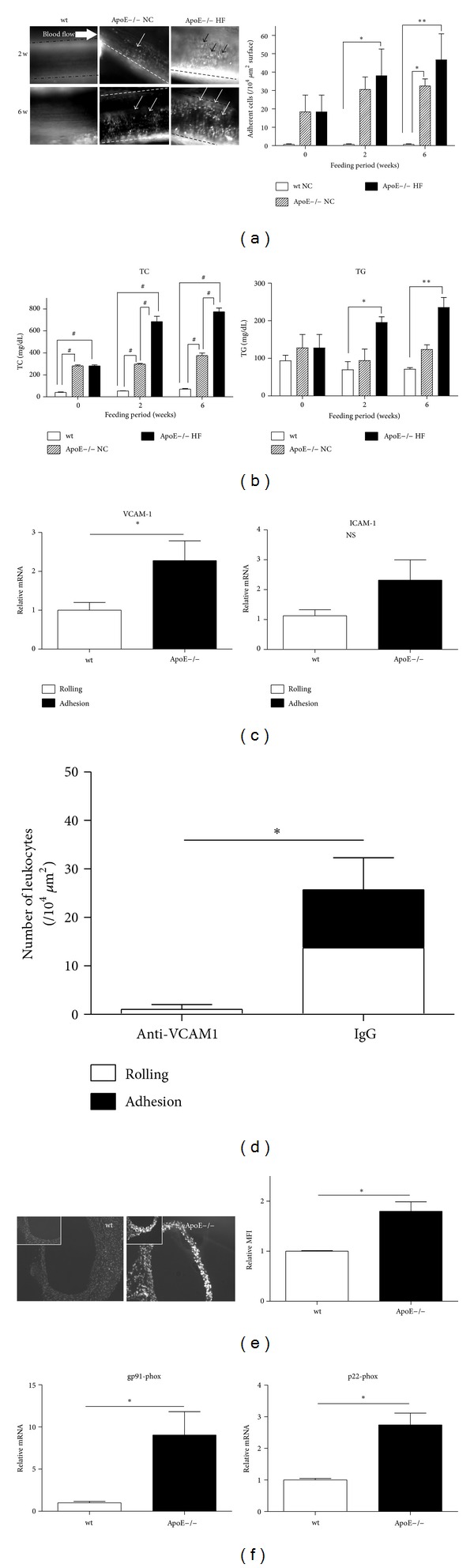
Leukocyte recruitment in the femoral artery of ApoE−/− mice fed HF. (a) Snap-shot microphotographs of the intravital microscopy system (IVM) (left, ×200) and their quantification (right graph) (*P < 0.05, **P < 0.01). Arrow heads in microphotographs show adherent leukocytes in the femoral artery. (b) Total cholesterol level and triglyceride level in plasma ofApoE−/− mice fed NC or HF for 2 weeks or 6 weeks (*P < 0.0001). (c) Relative mRNA expression levels of VCAM-1 and ICAM-1 in aortas of wt and ApoE−/− mice (*P < 0.05). (d) Leukocyte recruitment in following blockade of VCAM-1 by antibody (*P < 0.05). (e) Microphotograph of the aorta of ApoE−/− mice and wt stained by DHE (×200). (f) mRNA expression of gp91-phox and p22-phox in aorta of ApoE−/− mice (*P < 0.05).

First we observed leukocyte recruitment to “noninjured” femoral arteries of wt or ApoE−/− mice fed NC. As shown in Figure 1(a), prominent leukocyte adhesion was observed as early as 6 weeks of age (0 weeks feeding) in ApoE−/− mice (31.67 ± 19.43/10^4^ *μ*m^2^ vessel surface, *n* = 3) and had increased at 12 weeks of age (6 weeks-feeding NC, 32.43 ± 3.91, versus wt *P* < 0.05, *n* = 3), whereas no adhesion was observed in femoral arteries of wt at any time points (2 w 0.0 ± 0.0, *n* = 3; 6 w 0.0 ± 0.0, *n* = 3, Figure 1(a)). The plasma levels of total cholesterol (TC) significantly increased in ApoE−/− mice fed NC when compared to wt mice at any time. The level of triglyceride (TG) did not change between ApoE−/− mice and wt mice (Figure 1(b)). Body weight, plasma glucose level, and blood pressure did not change between ApoE−/− mice and wt (data not shown). 

We examined the potential effect of a high-fat diet on leukocyte adhesion to the femoral artery. Since a high-fat diet alone failed to develop atherosclerosis in wild-type mice (data not shown), we utilized ApoE−/− mice and subjected them to a high-fat diet. Interestingly, the number of adherent cells in ApoE−/− fed HF did not increase statistically when compared with those obtained from ApoE−/− mice fed NC (HF2w, 38.00 ± 14.57, *n* = 3, versus NC2w *P* = 0.63; HF6w, 46.67 ± 14.19, *n* = 3, versus NC6w *P* = 0.21, Figure 1(a)). TC level significantly increased in ApoE−/− mice fed HF when compared to NC feeding, whereas plasma TG did not significantly increased in ApoE−/− mice fed HF when compared with NC. (*P < 0.05, **P < 0.01, ^#^ *P* < 0.0001, *n* = 3, Figure 1(b)). 

### 3.2. Adhesion Molecule Expression in Vasculature and Leukocytes

To understand the mechanisms that induced leukocyte adhesion, we first measured cell adhesion molecules expressed on the vasculature. The expression of VCAM-1 was significantly induced in ApoE−/− mice compared with wt (ApoE−/− mice, 2.28 ± 0.51 folds, *n* = 10, 8; versus wt, *P* < 0.05). Similar inductions of ICAM-1 were observed though they were not statistically significant (2.32 ± 0.68, *n* = 9, 7, Figure 1(c)). 

### 3.3. Effect of Anti-VCAM-1 Antibody in ApoE−/− Mice Fed HF

To examine the effect of VCAM-1 on leukocyte recruitment, we examined anti-VCAM-1 antibody. The anti-VCAM-1 antibody diminished leukocyte adhesive interaction compared with control IgG (anti-VCAM-1, 1.00 ± 1.73 recruitment leukocytes, *n* = 3; control IgG, 22.33 ± 6.77, *n* = 3 versus anti-VCAM-1, *P < 0.05, Figure 1(d)).

### 3.4. Oxidative Stress of Atherosclerotic Aorta

 We then examined oxidative stress in the vasculature. As shown in Figure 1(e), strong DHE-associated fluorescence was observed in the aortas from ApoE−/− mice when compared with that from wt mice. The relative mean fluorescence intensity (MFI) in aortas of ApoE−/− mice significantly increased when compared with wt mice (wt 1.00 ± 0.008, *n* = 3; ApoE−/− mice, 1.800 ± 0.109, *n* = 3, *P < 0.005, Figure 1(e)). Expressions of gp91-phox and p22-phox, components of NADPH oxidase were significantly upregulated in ApoE−/− mice (gp-91-phox, 9.04 ± 2.54 folds, p22-phox, 2.70 ± 0.37, *n* = 6; *P* < 0.05 versus wt, Figure 1(f)). 

### 3.5. Effect of Rosuvastatin on Leukocyte Recruitment in ApoE−/− Mice

**Figure 2:**
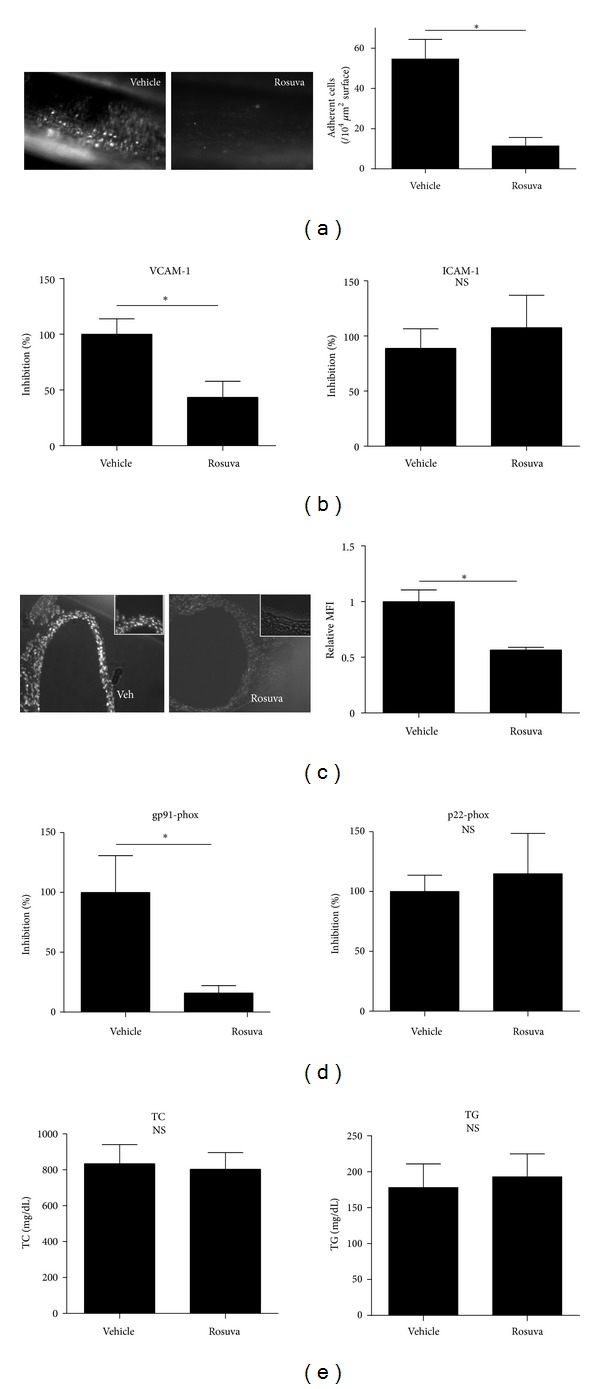
Effect of rosuvastatin on leukocyte recruitment to the femoral artery of ApoE−/− mice. ApoE−/− mice were administered HF with rosuvastatin for 6 weeks of age to 6 weeks. (a) Captured image of leukocyte interaction in a femoral artery treated with vehicle (left) and rosuvastatin (right). The number of adherent leukocytes was quantified by image analysis software. (b) Effect of rosuvastatin on expression levels of VCAM-1 and ICAM-1 (*P < 0.05). (c) Microphotographs of aortas in ApoE−/− mice treated with vehicle or rosuvastatin and stained by DHE (×200). (d) Effect of rosuvastatin on expression levels of gp91-phox and p22-phox in aortas of ApoE−/− mice (*P < 0.05). (e) Total cholesterol level and triglyceride level in plasma of HF-fed ApoE−/− mice treated with vehicle or rosuvastatin.

The anti-inflammatory role of rosuvastatin was examined in this model. As shown in Figure 2(a), rosuvastatin significantly reduced the number of adherent cells on the endothelium of the femoral artery of ApoE−/− mice (vehicle 54.63 ± 9.75/10^4^ *μ*m^2^ vessel surface, *n* = 8; rosuvastatin 11.43 ± 4.21, *n* = 7; *P* < 0.005). The expression level of VCAM-1 was significantly reduced by rosuvastatin treatment (43.43 ± 14.41% inhibition, *n* = 6; *P* < 0.05 versus vehicle), but the ICAM-1 level was not changed (113.14 ± 36.20, *n* = 6, Figure 2(b)). Furthermore, DHE-sensitive oxidative stress decreased in the aortas of ApoE−/− mice treated with rosuvastatin. Rosuvastatin significantly decreased relative MFI by DHE when compared with vehicle-treated aortas. (vehicle 1.00 ± 0.06, *n* = 3; rosuvastatin 0.57 ± 0.01, *n* = 3, *P < 0.005; Figure 2(c)). Rosuvastatin also significantly reduced the expression level of gp91-phox (15.95 ± 6.03% inhibition, *n* = 6; *P* < 0.05 versus veh). Meanwhile, p22-phox expression was not decreased by rosuvastatin (114.99 ± 36.76% inhibition, *n* = 6) (Figure 2(d)). No significant change in plasma TC or TG was observed with or without rosuvastatin treatment in ApoE−/− mice (Figure 2(e), *n* = 3).

### 3.6. Effect of Rosuvastatin on the Atherosclerotic Lesion

**Figure 3:**
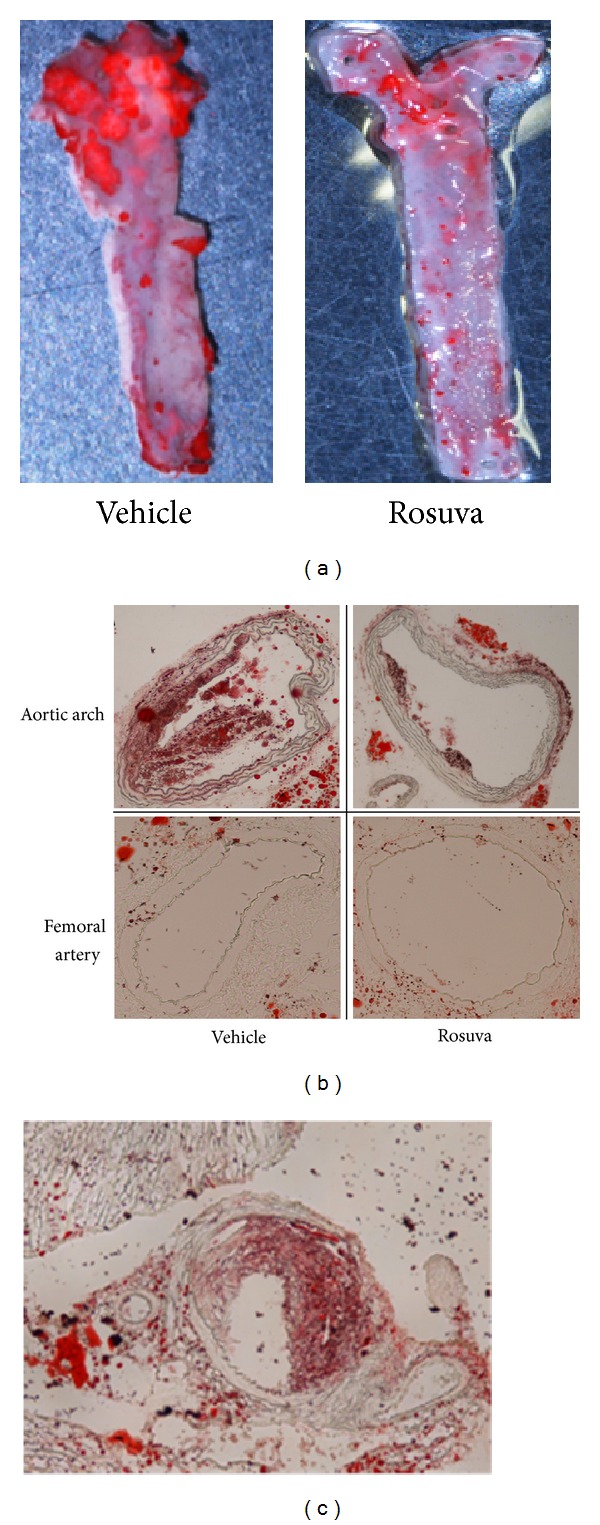
Effect of rosuvastatin on formation of atherosclerotic plaque. (a) Oil red-O staining of aortas of ApoE−/− mice treated with or without rosuvastatin. (b) Oil red-O staining at aortic arch and femoral artery treated with rosuvastatin or vehicle (×200). (c) Oil red-O stain for femoral artery of ApoE−/− mouse at 52 weeks of age.

The size of atherosclerotic lesions at the aortic arch was significantly decreased in ApoE−/− mice treated with rosuvastatin when compared with those treated with vehicle (Figures 3(a) and 3(b) upper panel). In contrast, no lesion development was observed in the femoral artery of ApoE−/− mice at this time point (Figure 3(b)). Extended study confirmed the prominent atherosclerotic lesion at the femoral artery (Figure 3(c)).

### 3.7. Adoptive Transfer of MNC Treated with Rosuvastatin

**Figure 4:**
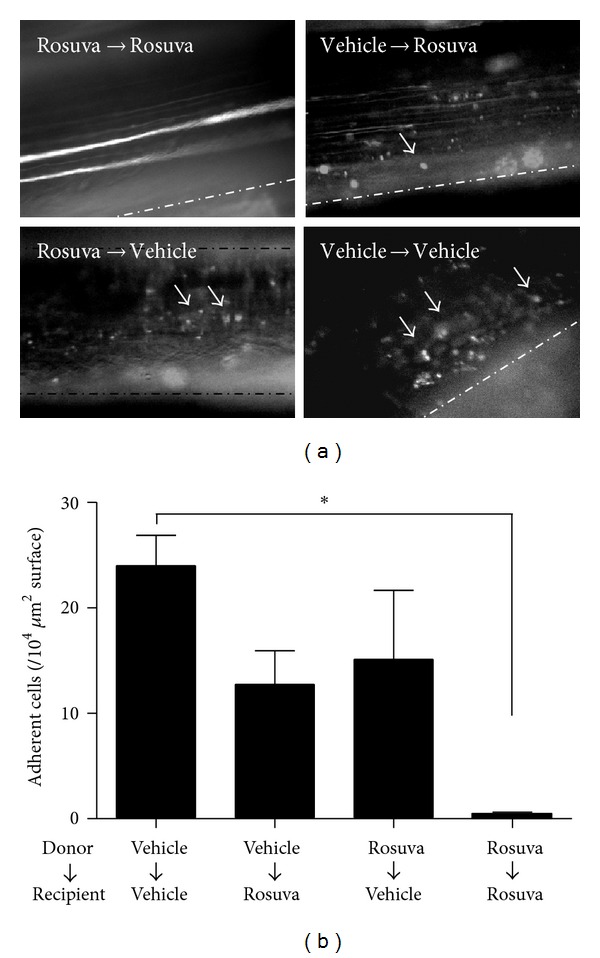
IVM analysis of adoptive MNC transfer experiments in ApoE−/− mice. Snapshot microphotographs (left) and their quantifications (right). MNC were isolated from ApoE−/− mice treated with or without rosuvastatin and injected into recipient ApoE−/− mice treated with or without rosuvastatin (*P < 0.05).

To determine whether rosuvastatin affects the leukocytes or the vascular tissues, we performed adoptive transfer of peripheral MNCs. MNCs from mice treated with vehicle were harvested, labeled ex vivo with Rhodamine 6G, and administered intravenously into recipient mice treated with vehicle (24.00 ± 2.89/10^4^ *μ*m^2^ vessel surface, *n* = 3). As shown in Figure 4, the leukocytes from donor mice adhered to the recipient femoral artery in a similar way to the endogenous leukocytes. In contrast, when MNCs prepared from mice with vehicle were infused into recipient mice treated with rosuvastatin, MNC recruitment was slightly decreased (12.75 ± 3.20, *n* = 4). Similarly, when MNCs prepared from mice with rosuvastatin were injected into recipient mice with vehicle, MNC recruitment was also slightly decreased (17.20 ± 8.62, *n* = 7). However, the magnitude of anti-adhesive effects was comparable among these two groups. When MNCs prepared from mice treated with rosuvastatin were injected into recipient mice treated with rosuvastatin, MNC recruitment in the recipient artery was significantly inhibited (0.40 ± 0.25, *n* = 5).

## 4. Discussion

In this study, we were able to observe leukocyte recruitment to non-injured femoral arteries of ApoE−/− mice as early as 6 weeks of age. Interestingly, pathological examination of the specimen revealed that there was no atherosclerotic lesion formation at this age. Whereas prominent atherosclerotic lesions were found in the same vascular region of the femoral artery at 52 weeks (Figure 3(c)). Thus, observation of the femoral artery at an early time point may be suitable for studying vascular inflammatory change in atherogenesis. To our knowledge, our data are the first to demonstrate that leukocyte recruitment to the luminal surface of the vasculature precedes atherosclerotic plaque formation in vivo. We also tried to examine a potential contribution of high-fat diet on early vascular inflammation observed in the femoral artery of ApoE−/− mice. Though previous study examined that a high fat diet significantly accelerate atherosclerosis lesion formation in ApoE−/− mice [26], we failed to detect a significant increase in the number of adherent leukocytes at 6 weeks after a high-fat diet. Potential qualitative differences of leukocyte adhesion such as distinct cell type recruited by NC and HF may affect these data observed in those treated with NC and high fat diet. These phenotypic differences in adherent leukocytes in the femoral artery will be examined in our future studies. Next, we examined the expression level of adhesion molecules in these mice. As shown in Figure 1(c), VCAM-1, but not ICAM-1 was upregulated in ApoE−/− mice. Further, antibody against VCAM-1 significantly blocks leukocyte adhesion to the femoral artery. These data strongly suggest a contribution of VCAM-1 in leukocyte adhesion in ApoE−/− mice. In good agreement with our data, Nakashima et al. [27] also reported enhancement of VCAM-1 expression in the aortic arch of ApoE−/− mice. As we demonstrated in Figure 1(e), oxidative stress is also accumulated in the vasculature of ApoE−/− mice. Lee et al. reported that VCAM-1 expression is closely related with oxidative stress via Sp1-dependent gene regulation [28]. Cayatte et al. found that inhibition of NADPH oxidase activity decreased atherosclerotic lesion areas in ApoE−/− mice via reduction of VCAM-1 expression [29]. Taking all together including ours, oxidative stress may play an important role to induce expression of VCAM-1 in ApoE−/− mice. In this study, expression levels of gp91-phox and p22-phox were increased in aortas of ApoE−/− mice. Sustained hyperlipidemia in ApoE−/− mice increases their systemic oxidative stress [30]. Our finding may point to a pivotal role of oxidative stress in connecting hyperlipidemia and vascular inflammation in vivo. We also confirmed an antiadhesive effect of rosuvastatin. The lipid-independent effect of statins has been focused in recent years and our groups confirmed mechanistic insights of antiadhesive effect of statin in vitro using physiological flow conditions [31]. Current data further strengthened our previous observation by using in vivo imaging system. Since serum cholesterol levels were not affected by rosuvastatin treatment (Figure 2(e)), our finding is not due to the improvement of hyperlipidemia in ApoE−/− mice. Rather, inhibition of oxidative stress, primarily reduction of gp-91phox, may play an important role in this process. This observation is in good agreement with previous studies [32, 33]. Adoptive transfer of MNC revealed that both vascular wall and leukocytes were target of rosuvastatin to reduce leukocyte recruitment, which exhibited comparison to own previous study using ARB in leukocyte adhesion in vivo, where leukocyte activation plays a dominant role [22]. 

## 5. Conclusion

 We were able to document leukocyte adhesion to the endothelium of the femoral artery in mice with dyslipidemia. The underlying mechanism seemed to involve oxidative stress and VCAM1 expression. Rosuvastatin abolished these phenomena via downregulation of gp91-phox, a component of NADPH oxidase. These results indicate that rosuvastatin has a protective effect against vascular inflammation and oxidative stress.
